# Discontinuous, biphasic, ontogenetic shifts in the metabolic allometry of aquatic animals?

**DOI:** 10.1242/bio.060317

**Published:** 2024-03-21

**Authors:** Gary C. Packard

**Affiliations:** Department of Biology, Colorado State University, Fort Collins, CO 80523, USA

**Keywords:** Allometry, Metabolic allometry, Biphasic allometry, Ontogenetic allometry, American eel, Spiny lobster

## Abstract

Several investigations in recent years have reported patterns of discontinuous, biphasic, loglinear variation in the metabolic allometry of aquatic animals. These putative shifts in pattern of allometry have been attributed to changes in the primary site for gas exchange from cutaneous to branchial as animals undergo ontogenetic changes in size, shape, and surface area. Because of the important implications of the earlier research with regard to both physiology and evolution, I re-examined data that purportedly support claims of discontinuous, biphasic allometry in oxygen consumption versus body size of American eels (*Anguilla rostrata*) and spiny lobsters (*Sagmariasus verreauxi*). I used ANCOVA to fit three different statistical models to each set of logarithmic transformations and then assessed the fits by Akaike's Information Criterion. The observations for both species were described better by a single straight line fitted to the full distribution than by a biphasic model. Eels, lobsters, and other aquatic animals undergo changes in shape and surface area as they grow, but such changes are not necessarily accompanied by changes in the pattern of metabolic allometry.

## Teaser

**Summary:** Allometric variation in metabolic rate is described by a straight line in a bivariate plot of logarithmic transformations of data for both American eels and spiny lobsters.

## INTRODUCTION

A recent investigation by Glazier and associates reported a pattern of discontinuous, biphasic, loglinear scaling of metabolic rate versus body mass in American eels (*Anguilla rostrata*), with the line fitted to observations for 30 juveniles having a lower slope (and higher intercept) on the logarithmic scale than the line fitted to observations for 30 subadults (Forlenza et al., 2022). This putative shift in pattern of metabolic allometry was attributed to a change in the primary site for gas exchange from cutaneous to branchial as animals underwent ontogenetic changes in size, shape, and surface area. However, conclusions from the investigation were quickly challenged on grounds that the statistical analysis of data from the study was compromised by a single, influential outlier in the form of a juvenile eel having an unusually high rate of oxygen consumption for an animal of its size and that the full distribution exclusive of the outlier (*n*=59) is well described by a single straight line (Packard, 2023). The challenge itself was subsequently rebutted by Glazier et al. (2023) in a response that included a new analysis in which body length for the same 60 eels was used, instead of body mass, as the measure of size. The new analysis using body length yielded the same general outcome as the initial analysis using body mass (i.e. discontinuous, biphasic, loglinear allometry), but the new analysis was not compromised by the appearance of an outlier. Thus, the full data set for the original analysis seemingly included one overly skinny juvenile, thereby creating the illusion of an outlier when mass was used as a measure of size (Glazier et al., 2023). The overall conclusion from the new analysis (with length as a measure of size) was the same as in the first analysis (with mass as the measure of size), namely, that a substantive, discontinuous shift in the pattern of metabolic allometry occurred as eels transitioned from juvenile to subadult stages. (Glazier et al., 2023).

The concept in question here is one of general importance (Hirst et al., 2014; Glazier et al., 2015): do ontogenetic changes in size and shape of aquatic animals affect sites and patterns of gas exchange in ways that elicit concomitant changes in patterns of metabolic allometry? Inasmuch as these coupled hypotheses are based on putative differences in patterns of metabolic allometry at different stages in development, it is imperative that the existence of different patterns be confirmed. Accordingly, I re-examined the new data for scaling of oxygen consumption versus body length in juvenile and subadult eels. I also re-examined data from a study on the scaling of oxygen consumption versus body mass in the early ontogeny of spiny lobsters (*Sagmariasus verreauxi*) because the investigation of spiny lobsters provided both rationale and perspective for the study of eels (Glazier et al., 2015). I find that claims for discontinuous, biphasic allometry in these two species actually have no support. In both instances, the data are described better on the logarithmic scale by a single straight line spanning the full range in size of the animals in question.

## RESULTS

### Scaling of metabolic rate versus body length in American eels

I recovered logarithmic transformations (base 10) for oxygen consumption and body length of eels from a supplement to the commentary by Glazier et al. (2023) and submitted them to an ANCOVA implemented in the Mixed Procedure in SAS 9.4 (see Table S1). The sample is comprised of observations from 30 juveniles and 30 subadults. The logarithm for oxygen consumption was the response variable in the analysis. Developmental stage was the predictor variable (a class variable), the logarithm for body length was the covariate, and an interaction between stage and log(body length) assessed the scaling slopes and intercepts for the two groups. The analysis was then run a second time without the interaction term to assess values for intercepts of lines having the same slope; and a third run retained only the covariate, that is, the logarithm for body length. The three models were evaluated and compared by Akaike's Information Criterion, with adjustments for sample size (AIC_C_; Burnham and Anderson, 2022).

**Fig. 1.**
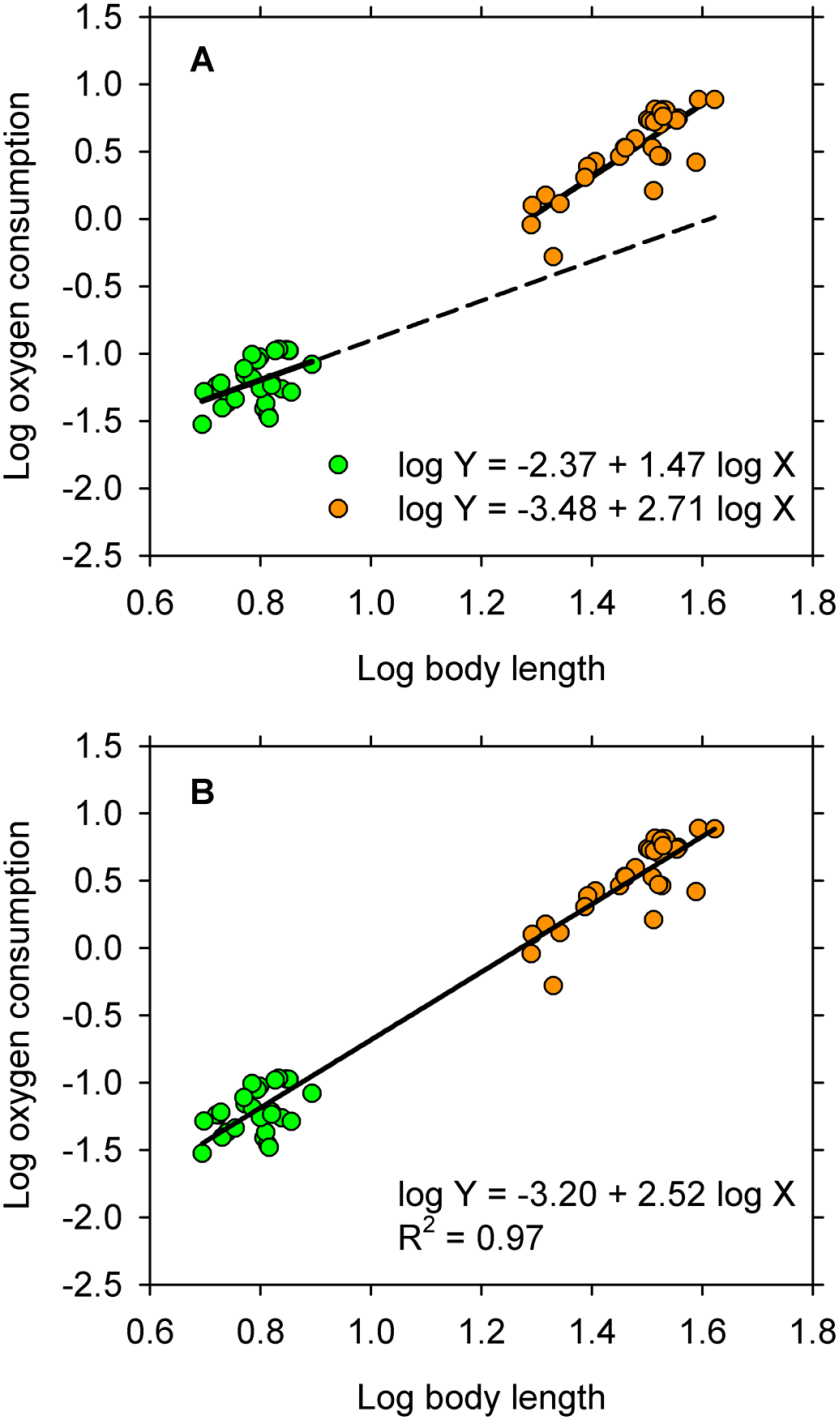
**Logarithmic transformations for oxygen consumption and body length of 30 juvenal and 30 subadult American eels.** Green, juveniles; orange, subadults. (A) Equations for straight lines fitted by the full model ANCOVA are identical to those reported by Glazier et al. (2023). The line fitted to observations for juveniles is extrapolated to illustrate the putative ontogenetic shift in metabolic allometry. (B) The straight line fitted by the reduced model ANCOVA describes pattern in the full sample.

The full ANCOVA (see Fig. S1) yielded the same equations for straight lines that are reported by Glazier et al. (2023) for juvenile and subadult eels (Fig. 1A). Likelihood values indicate that the full model also captured slightly more of the information in the data than did either of the alternative models, which were about equally efficient in this regard (Table 1). However, the higher recovery of information by the full model was achieved at the cost of additional parameters (five parameters in the full model versus four in the model lacking an interaction and three in the model for a single straight line). When a penalty was imposed for these additional parameters (Burnham and Anderson, 2022), the full model ANCOVA turned out to be no better by AIC_C_ than either of the other models considered here (Table 1). In other words, both the full model and the one lacking an interaction were overfitted to the data (Burnham and Anderson, 2022). Moreover, the probability for the interaction term in the full model is 0.06, and that for the group term is not compelling in either of the models in which this term appears (Table 1). Given this information, few applied statisticians would choose the full model with its all-important interaction term over the simplest of the candidate models, namely, the straight line fitted to the full distribution (Fig. 1B).

**Table 1.**
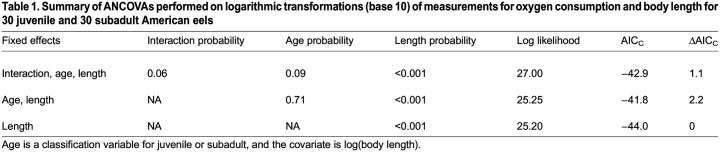
Summary of ANCOVAs performed on logarithmic transformations (base 10) of measurements for oxygen consumption and body length for 30 juvenile and 30 subadult American eels

### Scaling of metabolic rate versus body mass in spiny lobsters

**Fig. 2.**
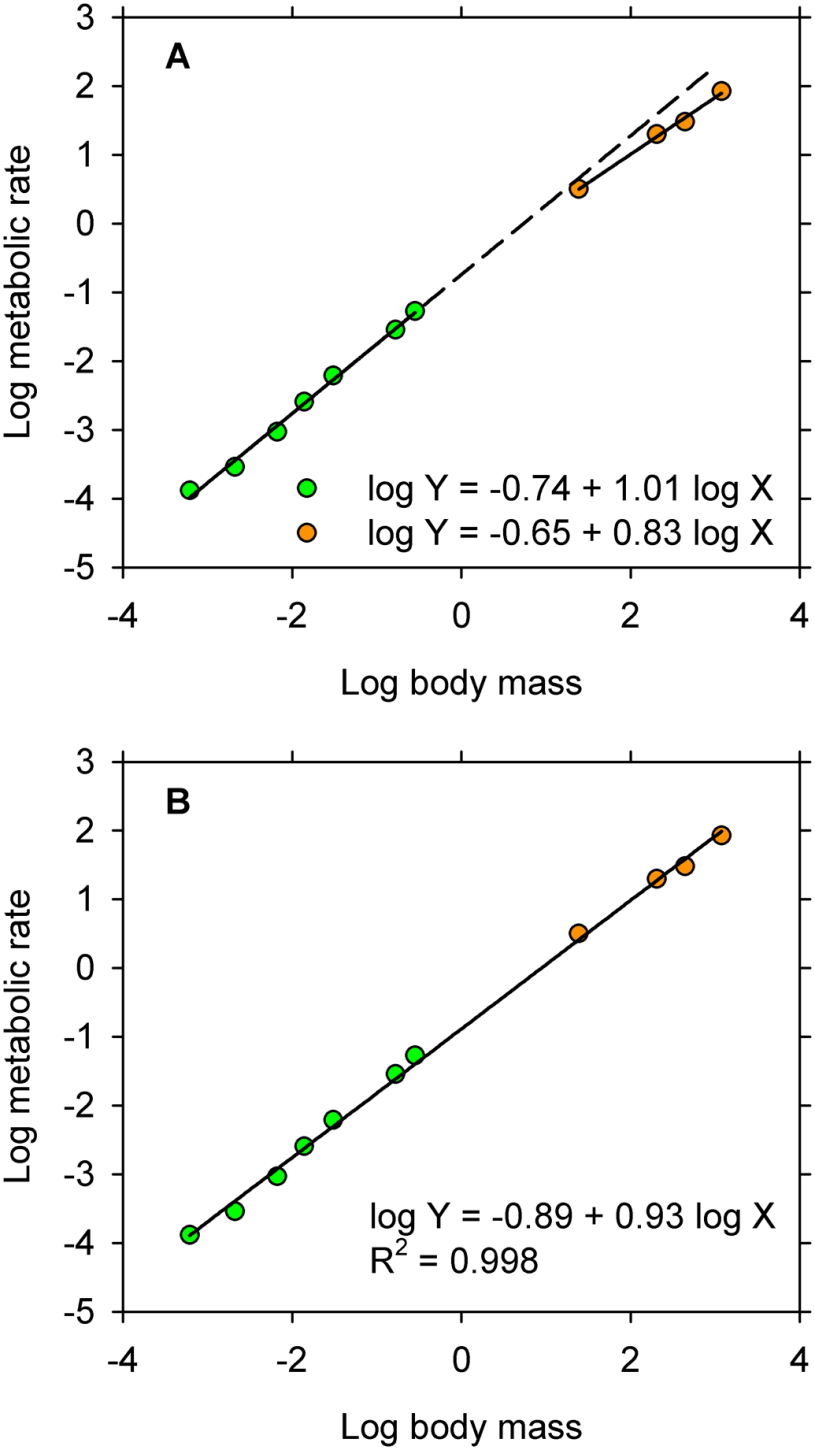
**Logarithmic transformations of oxygen consumption and body mass for juvenal spiny lobsters.** Each point represents the mean for 4–11 individuals. Green, larvae; orange, juveniles. (A) Equations for straight lines fitted by the full model ANCOVA are close approximations to the equations reported by Glazier et al. (2015). The line fitted to observations for juveniles is extrapolated to illustrate the putative ontogenetic shift in metabolic allometry. (B) The straight line fitted by the reduced model ANCOVA describes pattern in the full sample.

I used WebPlotDigitizer (https://automeris.io/WebPlotDigitizer/index.html) to capture logarithmic transformations for oxygen consumption and body mass in larval and juvenal spiny lobsters from Fig. 3B in the article by Glazier et al. (2015). Each observation in the figure is the mean for 4–11 measurements, so variability in individual responses cannot be assessed. The observations were first displayed on a bivariate graph (Fig. 2A) and then studied by ANCOVA using the same protocol that was used in the preceding example.

The interaction term in the full model ANCOVA is statistically significant by contemporary standards (Table 2), and values for likelihood indicate that the full model captures more of the information in the data than is captured by either of the alternative models (Table 2). On the face of it, the observations for larvae and juveniles appear to follow different trajectories (Fig. 2A). However, the relatively high likelihood for the full model was achieved at the cost of additional parameters. When a penalty for extra parameters is applied by AIC_C_ (Burnham and Anderson, 2022), it becomes apparent that the full model is overfitted (Table 2) and that the best model in the pool of candidates is the straight line describing the full distribution (Fig. 2B). The significant interaction in the full model is a spurious outcome of overfitting the model to small samples of means: seven for larvae and four for juveniles (Anderson et al., 2001). As in the preceding example, the best model in the pool of candidate models is that for a straight line spanning the full range in body size.

**Table 2.**
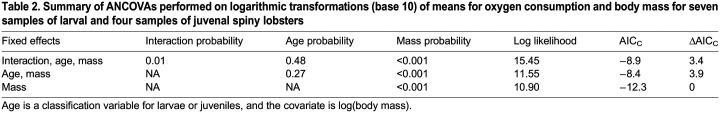
Summary of ANCOVAs performed on logarithmic transformations (base 10) of means for oxygen consumption and body mass for seven samples of larval and four samples of juvenal spiny lobsters

## DISCUSSION

Statistical analyses performed in the several studies by Glazier and associates yielded intercepts and slopes for regression models fitted to data for different age groups but failed to provide explicit tests of significance for differences between the parameters being compared. The authors assessed the importance of parameters indirectly by computing 95% confidence intervals about fitted values for the slope (intercept) for each line and by then determining whether the fitted value for slope (intercept) in one equation was within (outside) the confidence interval for the corresponding slope (intercept) in the other equation (Glazier, 2021). When neither of the slopes (intercepts) was included within the confidence interval for the other slope (intercept), the slopes (intercepts) for two equations were declared to be statistically different (Glazier, 2021).

The aforementioned approach to analysis suffered from two substantive problems. First, the protocol resulted in the fitting of one statistical model to the data (e.g. as in full ANCOVA with an interaction term): alternative models were not considered. However, when alternative models are considered (as in the current investigation), it becomes apparent that models with different slopes and intercepts for the different age groups provide overly complicated and misleading descriptions for pattern in the data. And second, comparing pairs of fitted values for slope (intercept) using confidence intervals as a guide is a generally unreliable method for assessing statistical significance (Sokal, 1965; Schenker and Gentleman, 2001; Wright et al., 2019). No claim for statistical significance can be sustained when confidence intervals for the parameters in question are broadly overlapping, as they are in all the investigations cited here (Glazier, 2021; Forlenza et al., 2022; Glazier et al., 2023).

The original analysis of allometric variation in oxygen consumption versus body mass in American eels (Forlenza et al., 2022) was compromised by a single, influential outlier (Packard, 2023). When the outlier was removed and the remaining data were examined by ANCOVA, the best model in the pool of candidate models was for a straight line fitted to the full range of observations in the data set (Packard, 2023). This same overall result is reported here for the scaling of metabolic rate versus body length in eels and for the scaling of metabolic rate against body mass in spiny lobsters (Tables 1 and 2). There is no support for a pattern of discontinuous, loglinear allometry in any of the data sets. While growth by both eels and lobsters doubtless elicits changes in surface area and sites for gas exchange, these changes have no discernable effect on patterns of metabolic allometry. Concepts that are based on putative patterns of discontinuous, biphasic variation in the metabolic allometry of aquatic animals need to be re-examined in light of the current findings.

## Supplementary Material

10.1242/biolopen.060317_sup1 Supplementary information
