# Supplementary material for: Discontinuous, biphasic, ontogenetic shifts in the metabolic allometry of aquatic animals?
Source: Biol Open. 2024 Mar 21;13(3):bio060317. doi: 10.1242/bio.060317 (PMC10979510; doi:10.1242/bio.060317)
Supplement: Supplementary information [file biolopen-13-060317-s1.pdf]

```
data mydata;  /**   Glazier eels full data set   **/
input X2    X     Z     Y;  /** X2=age (0=juvenile, 1=subadult), X=log
length, Z = log mass, Y= log MR **/
datalines;

0.0000      0.8388      -0.3468     -1.2625
0.0000      0.7987      -0.4815     -1.0271
0.0000      0.8062      -0.6990     -1.4095
0.0000      0.8561      -0.3665     -1.2883
0.0000      0.8136      -0.6021     -1.4599
0.0000      0.8096      -0.5086     -1.3690
0.0000      0.7767      -0.7447     -1.1409
0.0000      0.7945      -0.5686     -1.0496
0.0000      0.8470      -0.4202     -0.9726
0.0000      0.6946      -1.0000     -1.5258
0.0000      0.8195      -0.4318     -1.2156
0.0000      0.7846      -0.4559     -1.0074
0.0000      0.7235      -1.5229     -1.2427
0.0000      0.8519      -0.3188     -0.9788
0.0000      0.7723      -0.6990     -1.1574
0.0000      0.8513      -0.4202     -0.9768
0.0000      0.7210      -0.9586     -1.2388
0.0000      0.7853      -0.6990     -1.1859
0.0000      0.8000      -0.6576     -1.2593
0.0000      0.8932      -0.2218     -1.0805
0.0000      0.7396      -1.0000     -1.3685
0.0000      0.7308      -0.7447     -1.4043
0.0000      0.7701      -0.6576     -1.1113
0.0000      0.8331      -0.3979     -0.9694
0.0000      0.6981      -1.0458     -1.2851
0.0000      0.7284      -0.5686     -1.2210
0.0000      0.8267      -0.5376     -0.9802
0.0000      0.7543      -0.8539     -1.3378
0.0000      0.8162      -0.5528     -1.4804
0.0000      0.8202      -0.4318     -1.2365

1.0000      1.3160      0.9652      0.1750
1.0000      1.5119      1.8298      0.2092
1.0000      1.4065      1.3458      0.4244
1.0000      1.5092      1.8188      0.5286
1.0000      1.3927      1.2567      0.3882
1.0000      1.3874      1.3674      0.3065
1.0000      1.5888      2.0787      0.4189
1.0000      1.5224      1.8382      0.6994
1.0000      1.5011      1.7738      0.7386
1.0000      1.4594      1.5083      0.5298
1.0000      1.3304      1.1523      -0.2808
1.0000      1.5933      2.0395      0.8859
1.0000      1.5263      1.8698      0.4627
1.0000      1.4502      1.5843      0.4635
1.0000      1.5563      1.9791      0.7468
1.0000      1.4786      1.7024      0.5949
1.0000      1.5145      1.7259      0.8134
1.0000      1.2900      1.0569      -0.0429
1.0000      1.5211      1.8814      0.4691
1.0000      1.4609      1.5821      0.5274
1.0000      1.6222      2.1329      0.8853
```

Biology Open • Supplementary information

```
1.0000      1.5289      1.8463      0.8109
1.0000      1.5340      1.8274      0.8084
1.0000      1.2923      1.0492      0.0999
1.0000      1.5051      1.7993      0.7264
1.0000      1.5132      1.8319      0.7196
1.0000      1.5250      1.8537      0.7974
1.0000      1.5539      1.9074      0.7333
1.0000      1.5289      1.8797      0.7598
1.0000      1.3424      1.1875      0.1126
;

/*ods graphics off;*/



proc mixed data=mydata method = ML;
class X2;
model Y = X2 X X2*X/ solution residual;
run;

proc mixed data=mydata method = ML;
class X2;
model Y = X2 X/ solution residual;
run;

proc mixed data=mydata method = ML;
class X2;
model Y = X/ solution residual;
run;
```

Fig. S1. SAS Output

Table S1. SAS Code

The SAS System

The Mixed Procedure

Model Information	
Data Set	WORK.MYDATA
Dependent Variable	Y
Covariance Structure	Diagonal
Estimation Method	ML
Residual Variance Method	Profile
Fixed Effects SE Method	Model-Based
Degrees of Freedom Method	Residual

Class Level Information		
Class	Levels	Values
X2	2	0 1

Dimensions	
Covariance Parameters	1
Columns in X	6
Columns in Z	0
Subjects	1
Max Obs per Subject	60

Number of Observations	
Number of Observations Read	60
Number of Observations Used	60
Number of Observations Not Used	0

Covariance Parameter Estimates	
Cov Parm	Estimate
Residual	0.02379

Fit Statistics	
-2 Log Likelihood	-54.0
AIC (Smaller is Better)	-44.0
AICC (Smaller is Better)	-42.9
BIC (Smaller is Better)	-33.6

Biology Open • Supplementary information

Solution for Fixed Effects						
Effect	X2	Estimate	Standard Error	DF	t Value	Pr > |t|
Intercept		-3.4752	0.4664	56	-7.45	<.0001
X2	0	1.1018	0.6480	56	1.70	0.0946
X2	1	0	.	.	.	.
X		2.7059	0.3155	56	8.58	<.0001
X*X2	0	-1.2342	0.6489	56	-1.90	0.0623
X*X2	1	0	.	.	.	.

Type 3 Tests of Fixed Effects				
Effect	Num DF	Den DF	F Value	Pr > F
X2	1	56	2.89	0.0946
X	1	56	41.45	<.0001
X*X2	1	56	3.62	0.0623

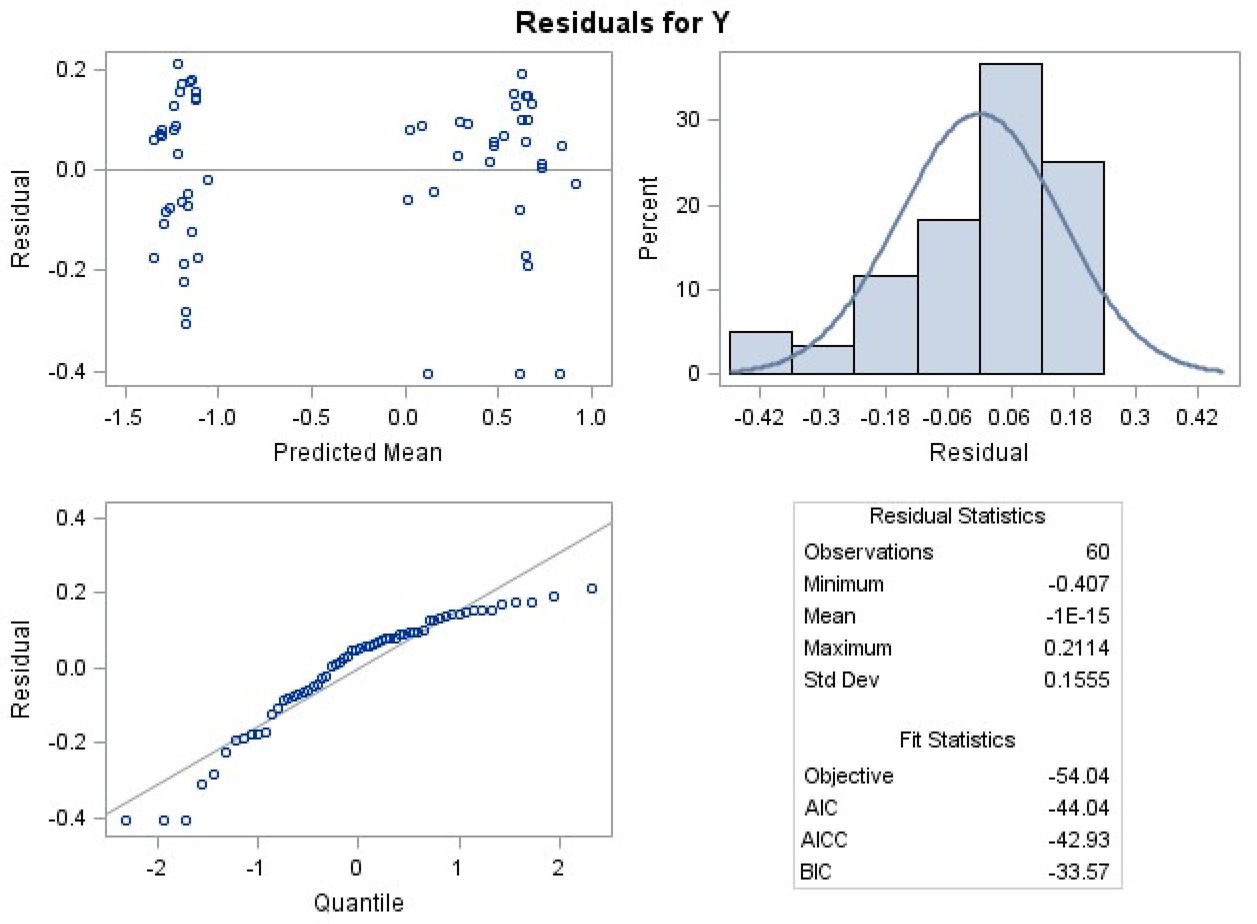

Residual Statistics	
Observations	60
Minimum	-0.407
Mean	-1E-15
Maximum	0.2114
Std Dev	0.1555

Fit Statistics	
Objective	-54.04
AIC	-44.04
AICC	-42.93
BIC	-33.57

Biology Open • Supplementary information

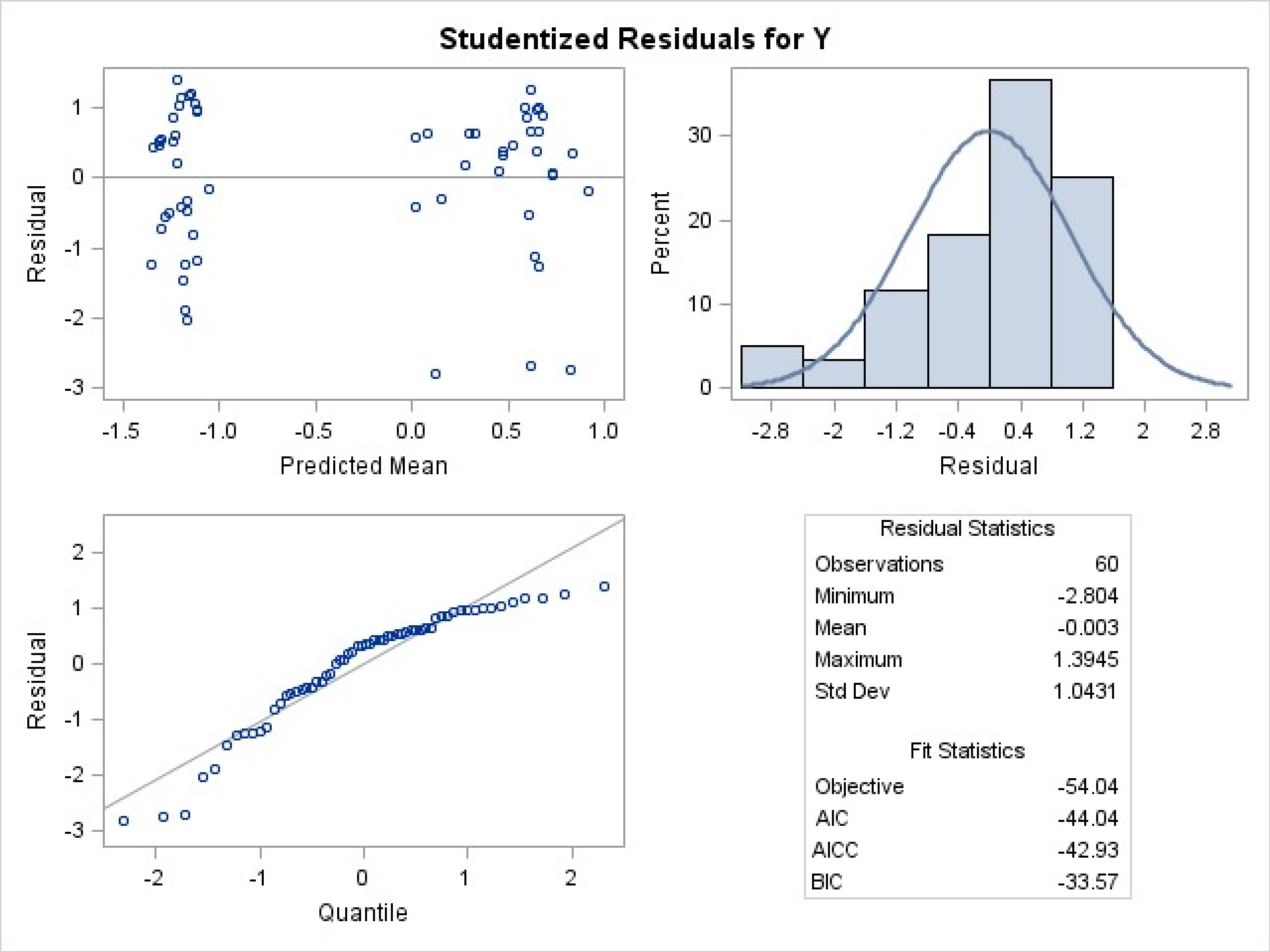
Studentized Residuals for Y
Residual
Predicted Mean
Percent
Residual
Quantile
Residual Statistics
Observations 60
Minimum -2.804
Mean -0.003
Maximum 1.3945
Std Dev 1.0431
Fit Statistics
Objective -54.04
AIC -44.04
AICC -42.93
BIC -33.57

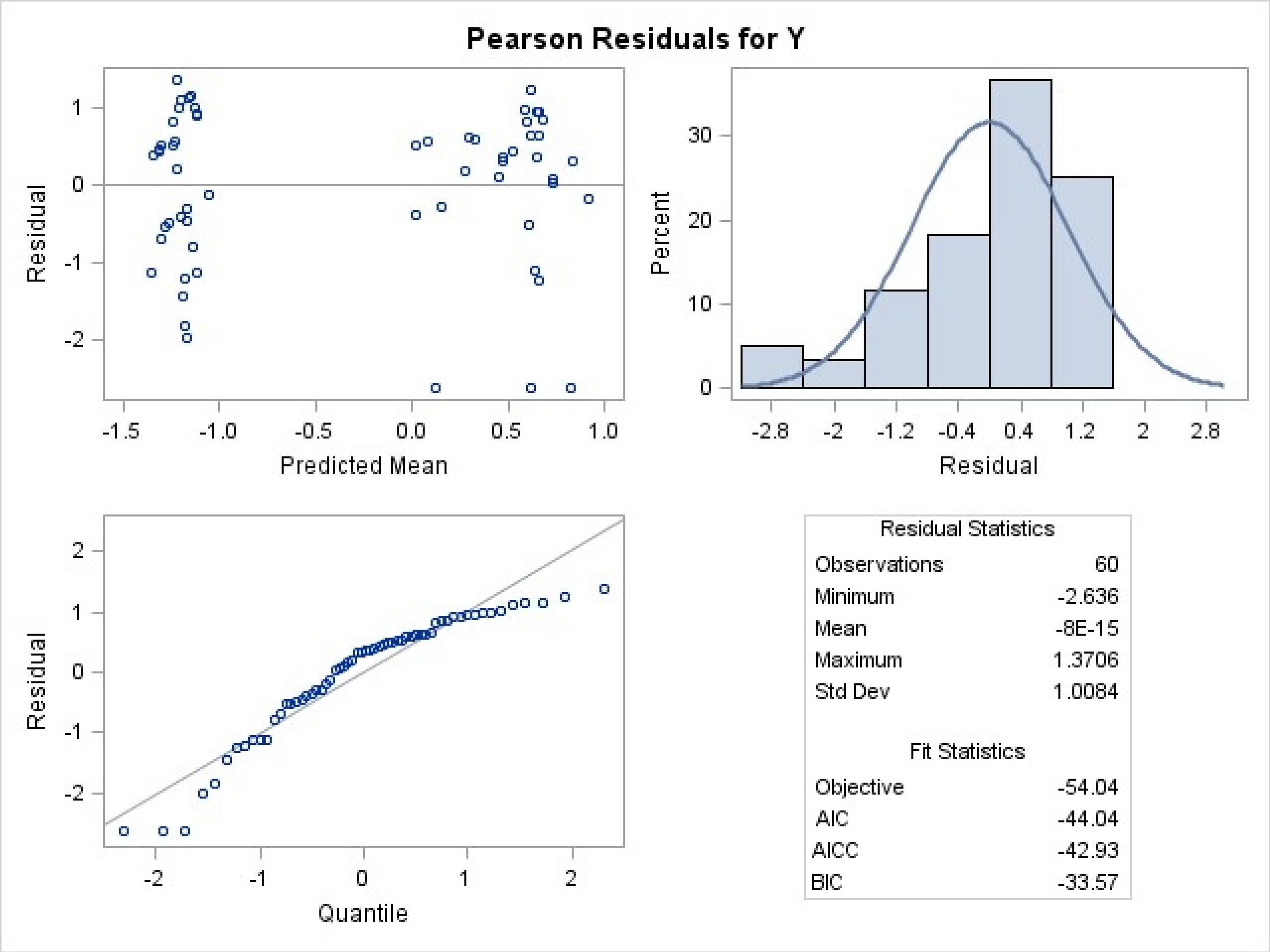
Pearson Residuals for Y
Residual
Predicted Mean
Percent
Residual
Quantile
Residual Statistics
Observations 60
Minimum -2.636
Mean -8E-15
Maximum 1.3706
Std Dev 1.0084
Fit Statistics
Objective -54.04
AIC -44.04
AICC -42.93
BIC -33.57

The SAS System

The Mixed Procedure

Model Information	
Data Set	WORK.MYDATA
Dependent Variable	Y
Covariance Structure	Diagonal
Estimation Method	ML
Residual Variance Method	Profile
Fixed Effects SE Method	Model-Based
Degrees of Freedom Method	Residual

Class Level Information		
Class	Levels	Values
X2	2	0 1

Dimensions	
Covariance Parameters	1
Columns in X	4
Columns in Z	0
Subjects	1
Max Obs per Subject	60

Number of Observations	
Number of Observations Read	60
Number of Observations Used	60
Number of Observations Not Used	0

Covariance Parameter Estimates	
Cov Parm	Estimate
Residual	0.02522

Fit Statistics	
-2 Log Likelihood	-50.5
AIC (Smaller is Better)	-42.5
AICC (Smaller is Better)	-41.8

Biology Open • Supplementary information

BIC (Smaller is Better)	-34.2

Solution for Fixed Effects						
Effect	X2	Estimate	Standard Error	DF	t Value	Pr > |t|
Intercept		-3.0447	0.4199	57	-7.25	<.0001
X2	0	-0.07500	0.1983	57	-0.38	0.7067
X2	1	0	.	.	.	.
X		2.4141	0.2839	57	8.50	<.0001

Type 3 Tests of Fixed Effects				
Effect	Num DF	Den DF	F Value	Pr > F
X2	1	57	0.14	0.7067
X	1	57	72.31	<.0001

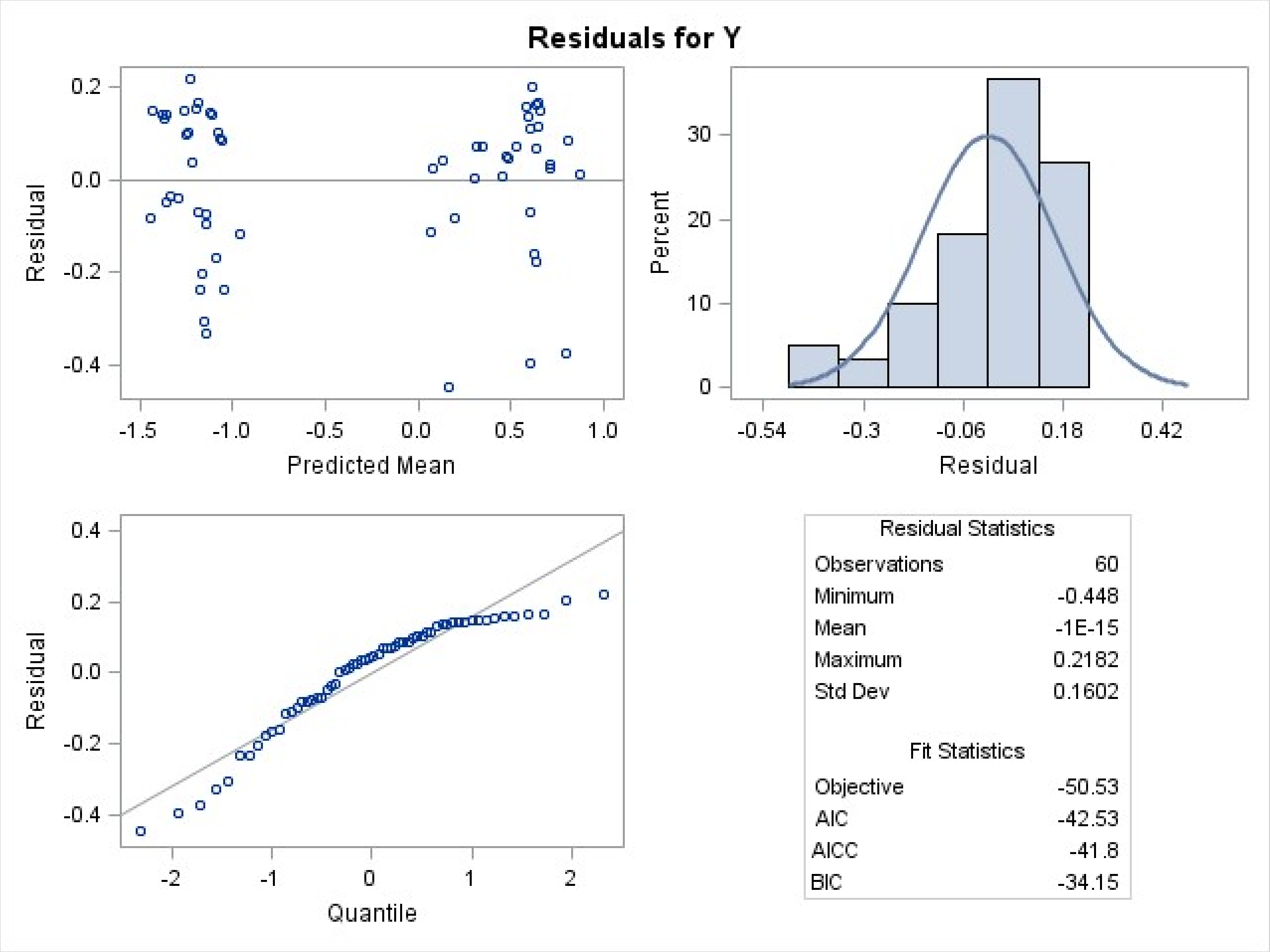

Biology Open • Supplementary information

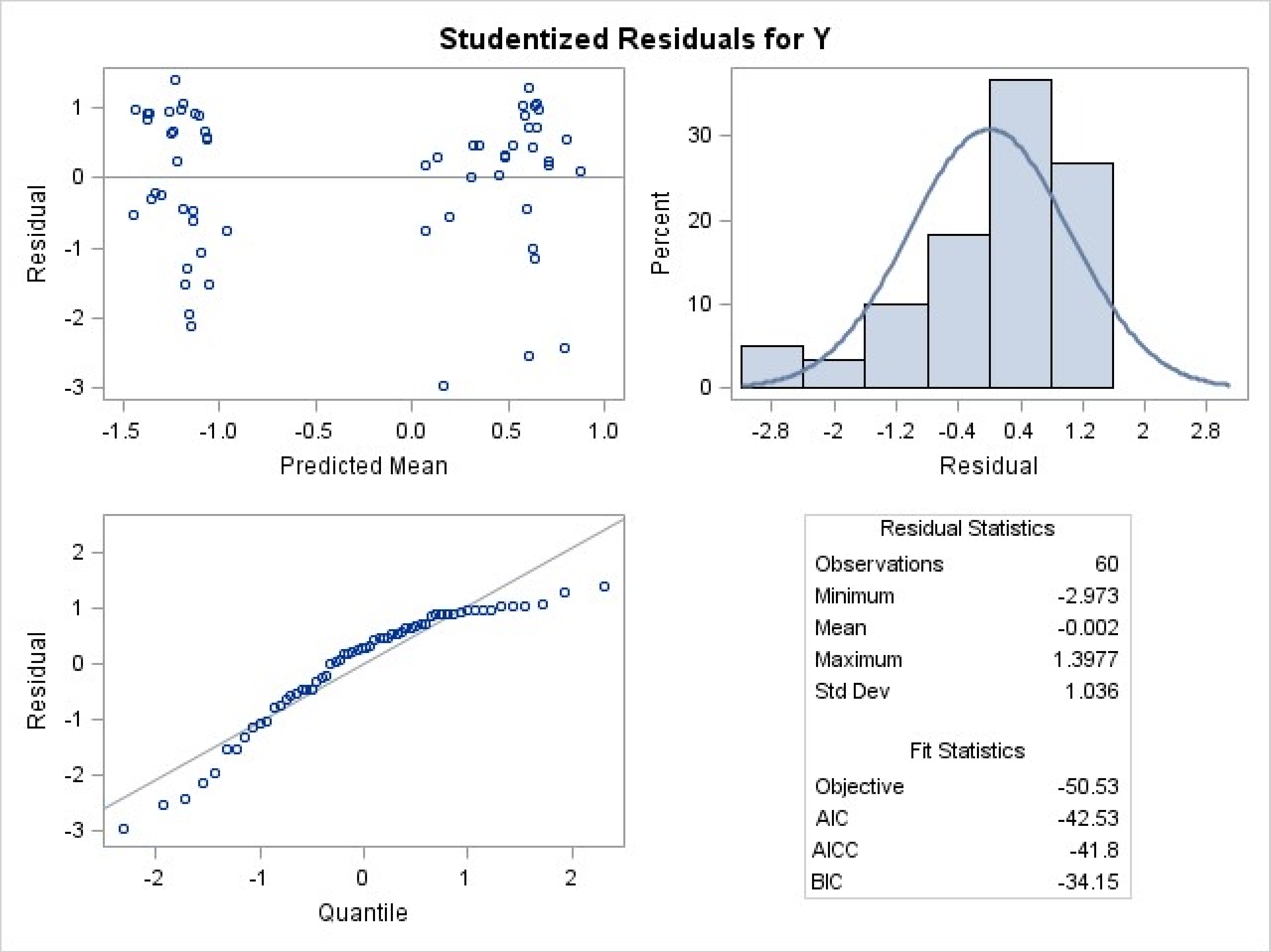
Studentized Residuals for Y
Residual
Predicted Mean
Percent
Residual
Residual
Quantile
Residual Statistics
Observations 60
Minimum -2.973
Mean -0.002
Maximum 1.3977
Std Dev 1.036
Fit Statistics
Objective -50.53
AIC -42.53
AICC -41.8
BIC -34.15

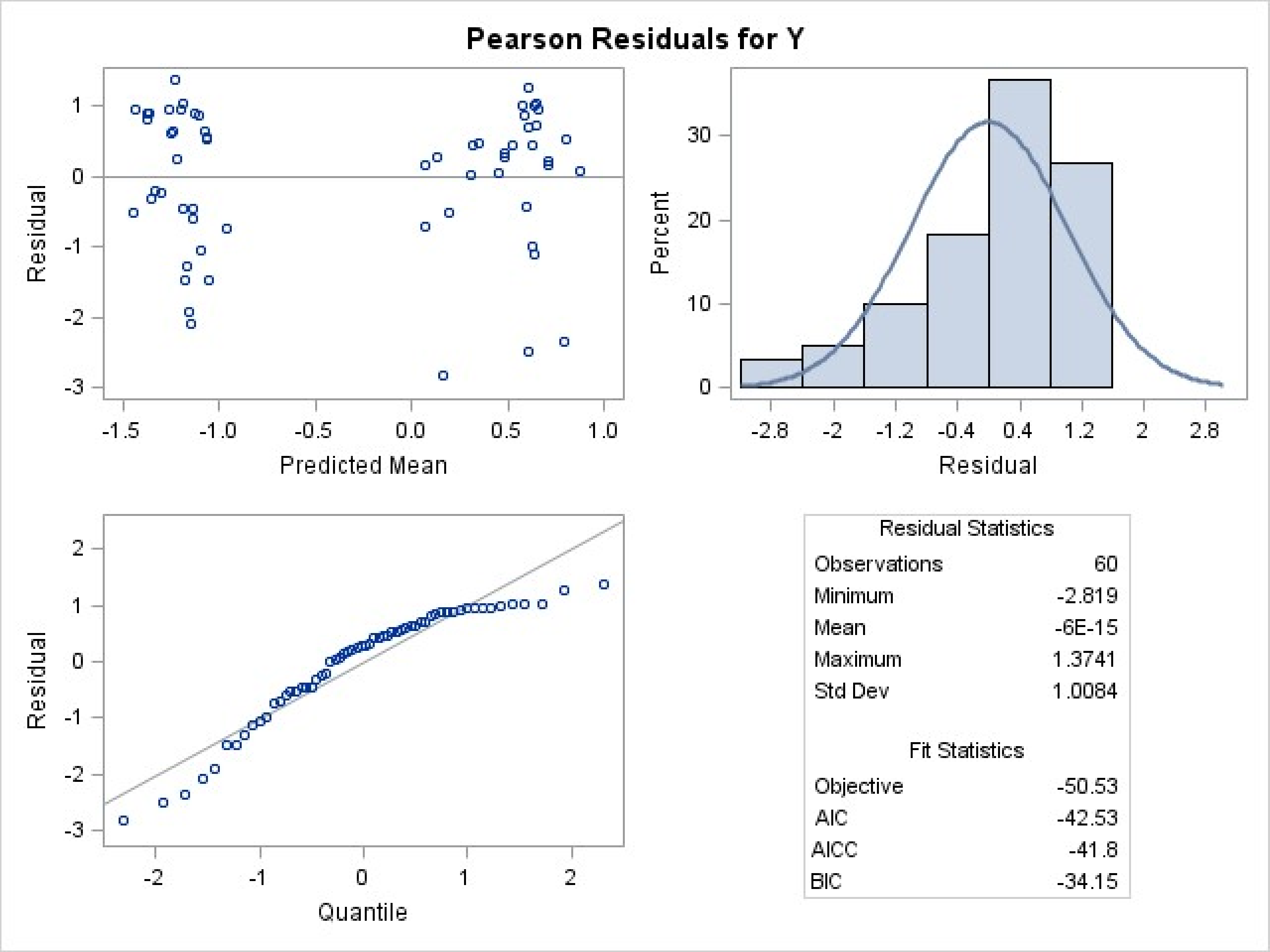

Biology Open • Supplementary information

The SAS System

The Mixed Procedure

Model Information	
Data Set	WORK.MYDATA
Dependent Variable	Y
Covariance Structure	Diagonal
Estimation Method	ML
Residual Variance Method	Profile
Fixed Effects SE Method	Model-Based
Degrees of Freedom Method	Residual

Class Level Information		
Class	Levels	Values
X2	2	0 1

Dimensions	
Covariance Parameters	1
Columns in X	2
Columns in Z	0
Subjects	1
Max Obs per Subject	60

Number of Observations	
Number of Observations Read	60
Number of Observations Used	60
Number of Observations Not Used	0

Covariance Parameter Estimates	
Cov Parm	Estimate
Residual	0.02528

Fit Statistics	
-2 Log Likelihood	-50.4
AIC (Smaller is Better)	-44.4
AICC (Smaller is Better)	-44.0

Biology Open • Supplementary information

BIC (Smaller is Better)	-38.1

Solution for Fixed Effects					
Effect	Estimate	Standard Error	DF	t Value	Pr > |t|
Intercept	-3.2013	0.06971	58	-45.92	<.0001
X	2.5191	0.05876	58	42.87	<.0001

Type 3 Tests of Fixed Effects				
Effect	Num DF	Den DF	F Value	Pr > F
X	1	58	1837.77	<.0001

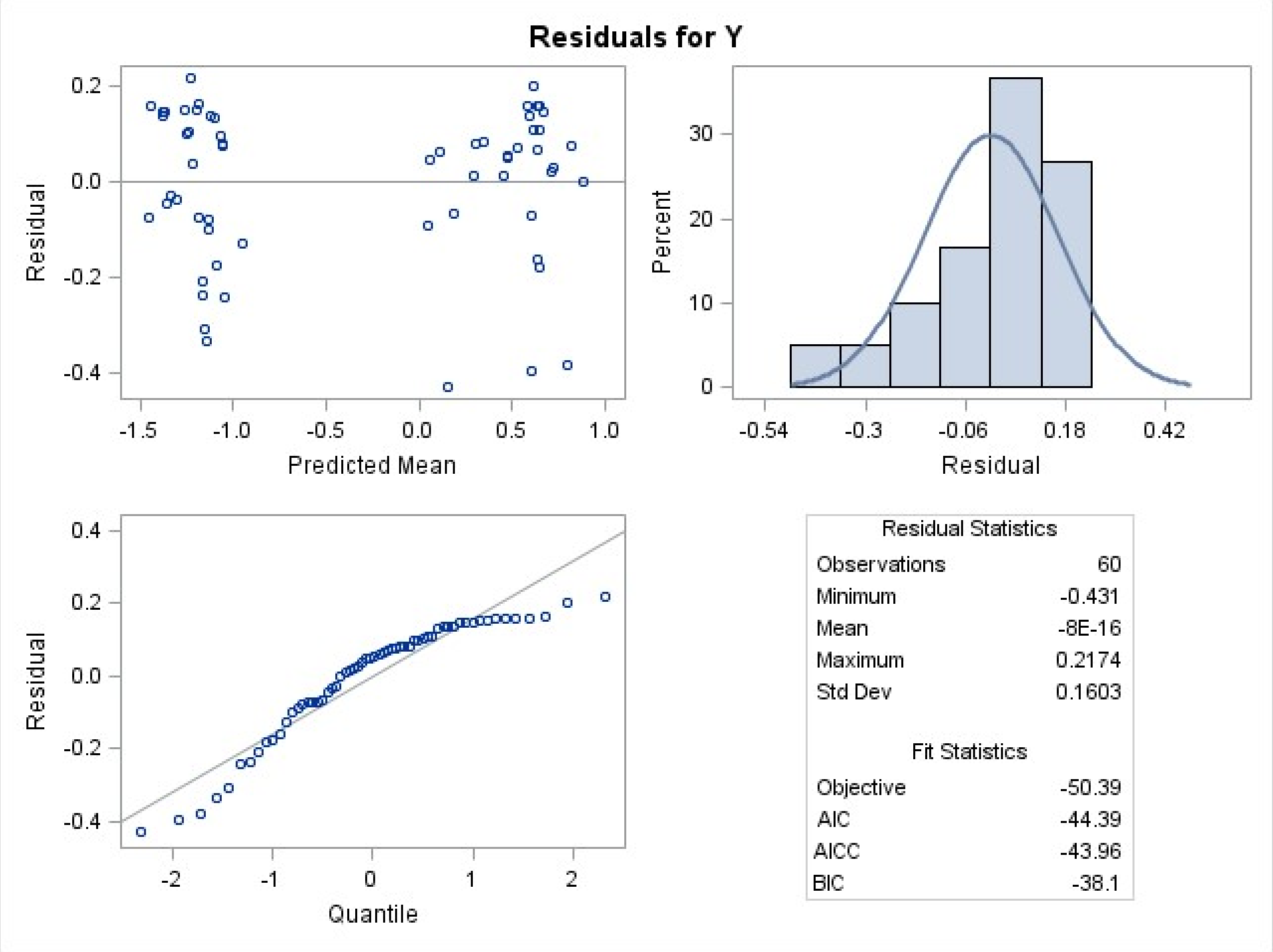

Biology Open • Supplementary information

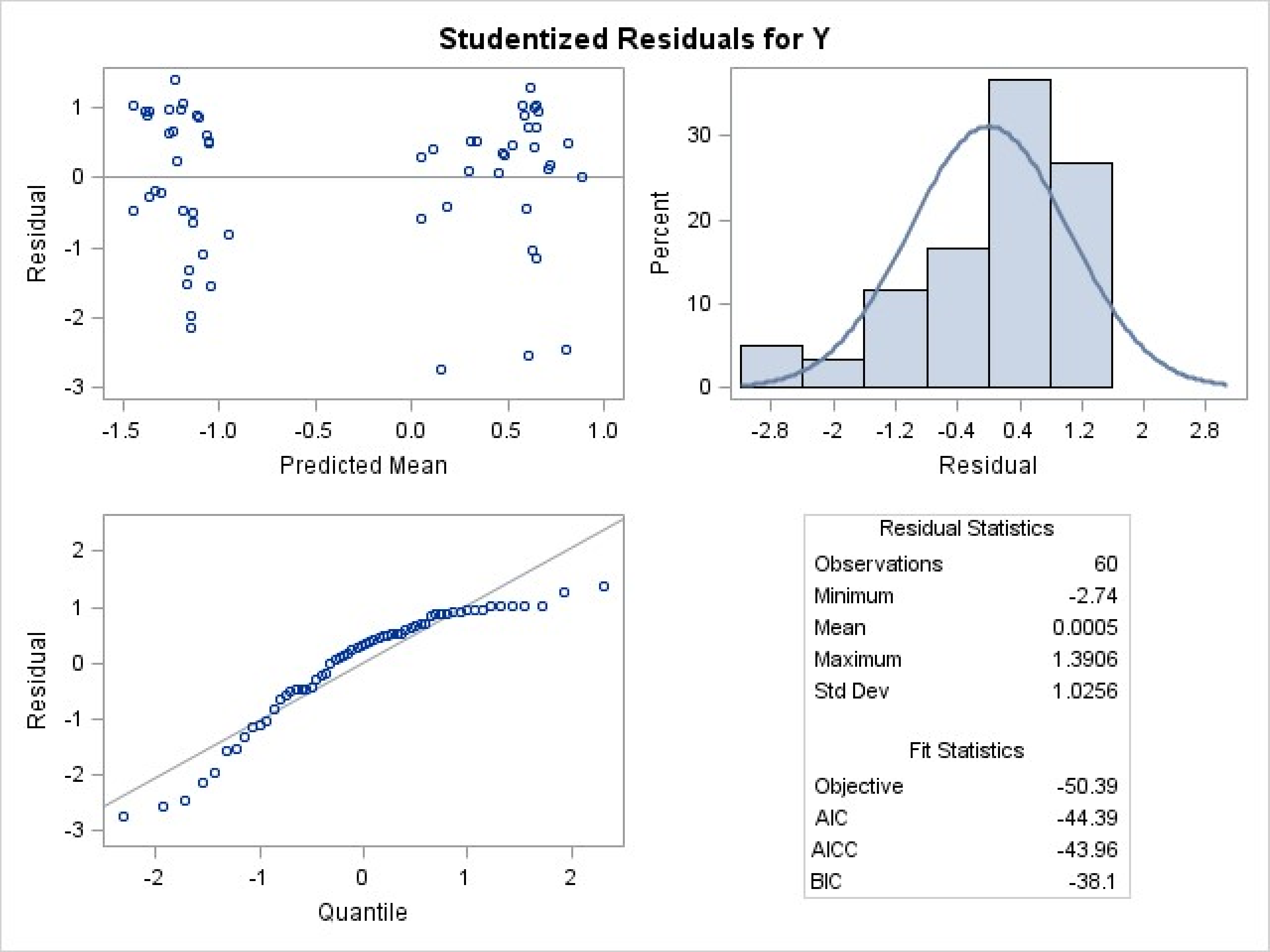
Studentized Residuals for Y
Residual
Predicted Mean
-1.5 -1.0 -0.5 0.0 0.5 1.0
1 0 -1 -2 -3
Percent
Residual
-2.8 -2 -1.2 -0.4 0.4 1.2 2 2.8
30 20 10 0
Residual
Quantile
-2 -1 0 1 2
2 1 0 -1 -2 -3
Residual Statistics
Observations 60
Minimum -2.74
Mean 0.0005
Maximum 1.3906
Std Dev 1.0256
Fit Statistics
Objective -50.39
AIC -44.39
AICC -43.96
BIC -38.1

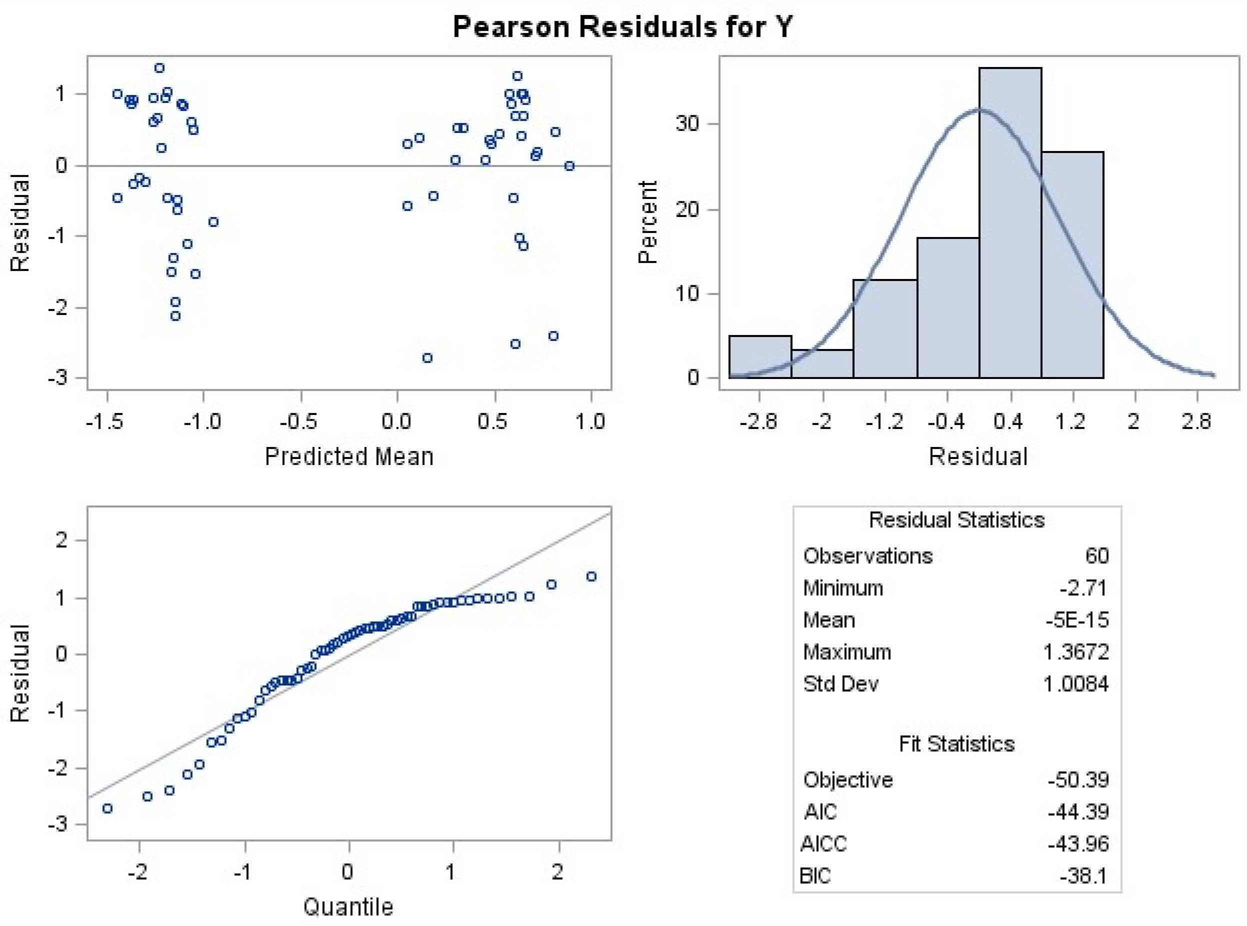

Residual Statistics	
Observations	60
Minimum	-2.71
Mean	-5E-15
Maximum	1.3672
Std Dev	1.0084

Fit Statistics	
Objective	-50.39
AIC	-44.39
AICC	-43.96
BIC	-38.1

Biology Open • Supplementary information
